# The “Dark Side” of Food Stuff Proteomics: The CPLL-Marshals Investigate

**DOI:** 10.3390/foods3020217

**Published:** 2014-04-17

**Authors:** Pier Giorgio Righetti, Elisa Fasoli, Alfonsina D’Amato, Egisto Boschetti

**Affiliations:** 1 Department of Chemistry, Materials and Chemical Engineering “Giulio Natta”, Politecnico di Milano, Via Mancinelli 7, Milano 20131, Italy; E-Mails: elisa.fasoli@polimi.it (E.F.); alfonsina.damato@chem.polimi.it (A.D.); 2 EB Consulting, Paris, France; E-Mail: egisto.boschetti@gmail.com

**Keywords:** animal foods, plant foods, alcoholic beverages, non-alcoholic beverages, aperitifs, proteomics, combinatorial peptide libraries

## Abstract

The present review deals with analysis of the proteome of animal and plant-derived food stuff, as well as of non-alcoholic and alcoholic beverages. The survey is limited to those systems investigated with the help of combinatorial peptide ligand libraries, a most powerful technique allowing access to low- to very-low-abundance proteins, i.e., to those proteins that might characterize univocally a given biological system and, in the case of commercial food preparations, attest their genuineness or adulteration. Among animal foods the analysis of cow’s and donkey’s milk is reported, together with the proteomic composition of egg white and yolk, as well as of honey, considered as a hybrid between floral and animal origin. In terms of plant and fruits, a survey is offered of spinach, artichoke, banana, avocado, mango and lemon proteomics, considered as recalcitrant tissues in that small amounts of proteins are dispersed into a large body of plant polymers and metabolites. As examples of non-alcoholic beverages, ginger ale, coconut milk, a cola drink, almond milk and orgeat syrup are analyzed. Finally, the trace proteome of white and red wines, beer and aperitifs is reported, with the aim of tracing the industrial manipulations and herbal usage prior to their commercialization.

## 1. Introduction

The present review aims at offering a panorama of what modern pre-fractionation technologies can achieve in detecting the low- to very-low abundance proteome in food stuff and beverages, an analyte fraction that is quite invisible even to the most sophisticated modern mass spectrometers (MS), whose sensitivity spans in general five orders of magnitude in relative concentrations of proteins present in a sample. Yet, in human biological fluids, such as plasma, such dynamic range can cover up to 12 orders of magnitude [1] and, in living cells, can span at least seven orders of magnitude. Thus, it is quite obvious that MS alone cannot cover efficiently the grounds and thus additional techniques would be needed to achieve the goal of (hopefully) a global coverage of any proteome. One such technique, which will be reviewed here with examples of what can be achieved in analysis of animal and “botanical” food stuff, as well as of non-alcoholic and alcoholic beverages, is definitely the combinatorial peptide ligand library (CPLL) technology that we have developed over the years, and which has now been taken at a level of maximum performance, as summarized in a recent book [2].

In a way it sounds preposterous that one should tackle such a detection of trace species in food stuff when, in the field of human nutrition, the problems we are facing are enormous, as today we have to feed a global population that has reached seven billion and is projected to reach nine billion by the year 2050 [3]. In the book here quoted [3], Brown depicts an almost apocalyptic scenario of land erosion, desertification, water shortage, dramatic increments of dangerous gases such as CO_2_ and NO and yields of cereals (the basic staple food for humans) rapidly reaching a limit. Were this not enough, there would be a dramatic imbalance of food distribution in the world population. About one third suffers from malnutrition, although in a peculiar way: 1.1 billion face starvation whereas 1.3 billion are either overweight or obese. One might think that the overweight population would be typical of the rich countries, such as USA (where indeed the phenomenon is reaching alarming proportions) but in reality it is widespread also in poor countries, since obese conditions can be attained by eating “junk food”. Yet, in well-to-do countries, the focus today is not on food quantity (the supermarkets seem to be bursting with food stuff) but on food quality, i.e., on how food can affect health and wellbeing of people, as illustrated below.

## 2. Nutraceutical and Functional Foods

The focus today is on detrimental to beneficial constituents in foods, as discussed in a recent issue of *Journal of Agricultural and Food Chemistry* [4,5,6]. This has led to a new science, called “nutraceutical”, a term that is a fusion of the words nutrition and pharmaceutical, thus emphasizing the potential therapeutic properties of foods. A new category of foods has thus emerged, the “functional food”, i.e., a food stuff that provides health or medical benefits beyond the basic nutrients it contains, including not just metabolites but proteins and peptides as well [7,8,9]. It is now well ingrained, in fact, that, whereas bad feeding habits are associated with, e.g., cardiovascular diseases, diabetes and even cancer, regular ingestion of “healthy foods” may prevent such diseases and/or mitigate them during onset and development [10]. The global nutraceutical market, defined as the cumulative sales of nutraceutical foods, beverages and supplements fortified with bio-active components, is now reaching a substantial segment of the food market. For instance, this market, which was worth $117.3 billion in 2007, has increased to 176.7 billion in 2013. A rather large proportion of these nutraceuticals is based on health claims, i.e., statements used on labels, in marketing or in advertising that health benefits can result from consuming a given food or from one of its components such as vitamins and minerals, fibre, and “probiotic” bacteria (which would ultimately form the population of gut microbiota) [11]. There are different types of health claims. For instance, statements that a food can help reinforce the body’s natural defenses or enhance learning ability are called “general function” claims. Examples also include claims on the reduction of disease risk and other substances that may improve or modify the normal functions of the body. Other types of claims are “nutrition claims”, which state or suggest that a food has particular beneficial nutritional properties. Examples comprise “low fat”, “source of omega-3 fatty acids” or “high in fibre”. In Europe alone, nutraceuticals have produced a stampede of applications to the regulatory agencies, for approval of their products, by mini- and start-up companies active (or entering) in this field. EFSA (European Food Safety Agency) has been flooded, only in the last few years, with 44,000 applications for approval of “potential” nutraceuticals, claiming a rainbow of biological activities. These applications have been gathered, analyzed and consolidated into about 4600 claims, of which, in 2012, only 222 were approved [12]. One wonders what the reason is for such a low promotion score. The vast majority of claims, indeed, lack the following basic information: (1) most of them do not even list or know which ones are the active and health beneficial ingredients, since they propose simply ground botanical extracts as such or only partially purified, whose chemical composition remains largely unknown; (2) by the same token, they lack *in vivo* demonstration of such health benefits, not just on humans, but not even on animal models. It is clear that tools for fully exploring their nature are sorely needed, especially those able to detect trace peptide/protein components, whose biological activities have to be demonstrated.

## 3. Separation Science at Work: Combinatorial Peptide Ligand Libraries (CPLL)

As stated above, CPLLs appear to be a unique tool for exploring the “dark side” of any proteome, due to their unique property of providing millions of affinity ligands able to find a partner in any protein species present in biological materials. It must be emphasized that in just about any biological specimen a small set of proteins (often as few as 20–30) are present in a large excess and could constitute, like in human sera, as much as 99% of the total protein mass. This would leave little room for sampling (and thus detecting) all other species present therein. A solution proposed already in 2002 by the Anderson’s lab is immuno-subtraction, i.e., preparation of affinity resins containing antibodies against the six most abundant proteins in sera (first extended to 12 and now to 20) [13]. Believed to permit access to low-abundance species [14], in reality it did not quite live up to expectations [15,16], for several reasons, among them the major issue being that much too little sample volume can be processed in a single sweep (barely 100 µL serum/plasma). Application of this methodology to plant proteomes, such as the immuno-depletion of RuBisCO, also did not lead to any major improvement [17].

**Figure 1:**
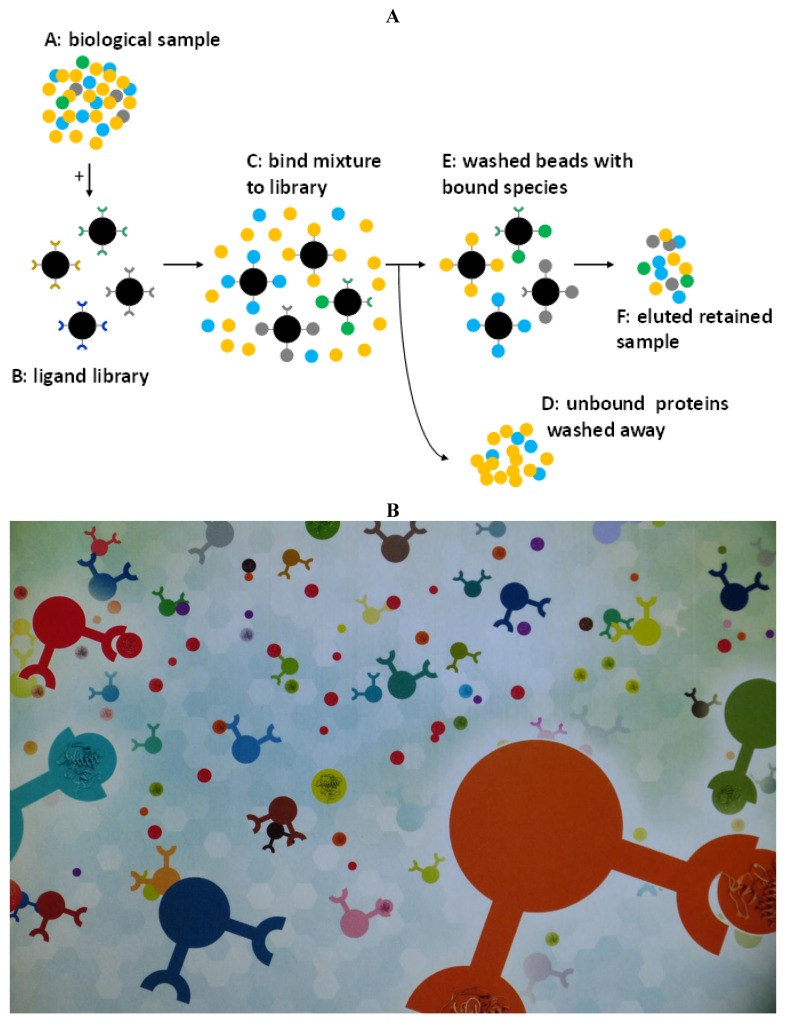
(**A**) Mechanism of action of combinatorial peptide ligand libraries. (**B**) Artistic rendering of combinatorial peptide ligand library (CPLL) beads with proteins docked on the hexapeptide baits.

The CPLL technique we have developed is immune from such drawbacks. Here is a brief description of the chemistry and mechanism of action of CPLLs. The material for the treatment of the biological sample is constituted of a mixture of discrete sorbents each of them carrying a different ligand (a hexapeptide). The number of individual species is extremely large. Each of them represents an item of a full mathematical combination of 15 amino acids built to a length of hexapeptides and obtained by combinatorial synthesis. Each discrete peptide is a potential ligand for one or more proteins from the sample. By loading the combined sorbent mix with a biological sample exceeding the binding capacity, each single affinity ligand will be saturated with corresponding protein partners and the excess will be found in the so-called flow-through. Abundant proteins will rapidly saturate the corresponding ligand while the rare proteins will continue to be adsorbed as long as the loading is increased, this process leading to their enrichment onto the beads. Once the sorbent mix is extensively washed in order to remove the excess proteins, the captured species are harvested by means of appropriate desorbing agents. All proteins are potentially present but their relative concentration is deeply changed with a strong compression of dynamic concentration range. The consequence of such a situation is the detection of many novel proteins (the low-abundance ones) as a result of (1) the annihilation of the signal suppression due to concentrated species (e.g., albumin in serum) and (2) the detection of very low concentration proteins that were below the detectability level prior to sample treatment. These two distinct phenomena, co-existing during the CPLL treatments, are very powerful when the loading is very large and when all captured proteins are harvested [2,18,19,20]. Figure 1A is a schematic representation of this sequence of steps and *modus operandi* of the beads, while Figure 1B is an artistic rendering of a bead suspension docking onto the respective partners (with a proviso, though: the beads do not have just two ligands but are coated with millions of ligands, all of the same hexapeptide type). We have applied the CPLL technique to analysis of food stuff and beverages for at least three main reasons:

1. to detect trace proteins/peptides exhibiting negative effects on health (e.g., allergens);
2. to detect trace proteins/peptides displaying positive effects on health (e.g., anti-microbial, anti-hypertensive and anti-oxidant activities);
3. to expose frauds in commercial food products and provide a proof of genuineness for “correct” commercial foods, as found in supermarkets.

The examples listed below are limited to analyses of foods we have performed over the years via the CPLL methodology. Moreover, we cannot possibly cover here the vast literature on plant and food proteomics, but we just mention two recent reviews (and references therein) covering this field quite extensively [21,22]. Special issues of different journals appear from time to time covering plant proteomics, including analysis of food and beverages. A recent one has been released by *Journal of Proteomics* [23]. Within this issue see, for instance, Boggess *et al.* [22], Nakamura *et al.* [24], Uvackova *et al.* [25], Agrawal *et al.* [26] and Ribeiro Demartini *et al.* [27].

## 4. Analysis of Animal Food Stuff

Already in 2008 we applied the CPLL technique to the analysis of the egg white proteome [28], soon followed by the exploration of egg yolk [29], the reason being that, in both cases, barely a handful of proteins was known, those of very high abundance. The first major exploration, though, performed via the most sophisticated MS equipment, had just described, prior to our investigations, 78 unique gene products, a quantum jump in respect to what was known up to that time [30]. Yet, in our case, even by using less powerful MS tools, we could almost double that value to 148 unique protein species, a non-negligible increment. Basically, the same events were repeated in regard to egg yolk. Just prior to our report, Mann and Mann [31] reported 115 unique gene products in the yolk plasma, whereas our findings have more than doubled this value to 255 unique protein species. These two manuscripts formed the core of an extensive review on the egg white and yolk interactomes [32]. Thus, these very first investigations proved that CPLLs had a lot to offer in terms of food analysis. Soon after we tackled the proteome of cow’s whey, not only for an in depth analysis, but also to detect “hidden” allergens that seemed to be responsible for unknown allergies suffered by babies that at that time were under observation at the Hôpital Cochin in Paris. Whereas comprehensive whey protein lists progressively increased in the last six years from 17 unique gene products to more than 100, our findings considerably expanded this list to a total of 149 unique protein species, of which 100 were not described in any previous proteomics study [33]. As an additional interesting result, a polymorphic alkaline protein was observed with a strong positive signal when blotted from an isoelectric focusing separation in gel and tested with sera of allergic patients. This polymorphic protein, found only after treatment with the peptide library, was identified as an immunoglobulin (Ig), a minor allergen that had been largely amplified. In terms of allergy to milk proteins, often quite deleterious for small babies, it had been known for quite some time that donkey’s milk was an excellent substitute for cow’s milk, allowing a normal and healthy growth for allergic children. So much so that two Italian pharmacists from Sicily, having observed that, opened a farm on the slopes of the Etna and started offering this milk with the brand name “Asilat” (asina is the Italian name of the she donkey), which has been a life-saver for thousands of Italian affected children. Yet, its composition remained largely unknown till our study with CPLLs [34]. By treating large volumes (up to 300 mL) of defatted, de-caseinized (whey) milk, we have been able to identify 106 unique gene products, by far the largest description so far of this precious nutrient. Due to poor knowledge of the donkey’s genetic asset, only 10% of the proteins could be identified by consulting the data base of *Equus asinus*; the largest proportion (70%) could be identified by homology with the proteins of *Equus caballus*. Among the species identified we could recognized also some epidermal growth factors, suggesting that Poppea’s (emperor Nero’s wife) habit of bathing in donkey’s milk, notwithstanding her scarce knowledge of proteomics, had some scientific merits! Our work on different types of milks deserves some further comments. In 2011, Le *et al.* [35] in an extensive investigation of both cow’s whey and colostrum, requiring a rather complex experimental set-up and analysis of several eluted fractions, reported a grand total of 293 unique proteins and thus stated that “the 149 characterized proteins may be a superficial representation of the proteome of bovine milk whey”. Soon after, Nissen *et al.* [36] delved again onto bovine colostrum and reported identification of 402 unique species, suggesting that the data of Le *et al.* [35] did not quite represent an in-depth exploration. Moreover, it just so happens that we are now analyzing cow’s colostrum by our (quite simple) CPLL methodology in collaboration with Prof. G. Aldini (University of Milano) in order to increase the knowledge of pharmaceutical activities related to low abundance proteins, of which we find a large number not reported before. So, at this point we are forced to state that their investigation [35] (notwithstanding the quite complex and elaborated experimental set-up) might be a “really superficial representation of this proteome”! All these collective data have helped Bislev *et al.* [37] to construct a Bovine Peptide Atlas comprising 1921 proteins at 1.2% false discovery rate (FDR) and 8559 distinct peptides at 0.29% FDR (with a caveat, though: this atlas comprises not only milk and colostrum, but a variety of bovine tissues, including hoof specimens). 

As an additional example of the application of CPLLs to food analysis, Di Girolamo *et al*. [38] recently investigated the proteome content of different unifloral honeys (from chestnut, acacia, sunflower, eucalyptus and orange) in order to see if any plant proteins present would allow the proteo-typing of these different varieties. It turned out that all proteins identified (except one, the enzyme glyceraldehyde-3-phosphate dehydrogenase) were not of plant origin but belonged to the *Apis mellifera* proteome. Among the total proteins detected (eight, but only seven as basic constituents of all types of honey), five belonged to the family of major royal jelly proteins 1–5, and were also the most abundant ones in any type of honey, together with α-glucosidase and defensin-1. It thus appears that honey has a proteome resembling the royal jelly proteome (but with considerably fewer species), except that its protein concentration is lower by three to four orders of magnitude as compared to royal jelly. The only other report available on the honey proteome could identify barely one protein [39]. In a way, this food stuff should be listed in the section below, but in reality it is a hybrid: although the origin is floral, the proteome is essentially of animal origin. We have also analyzed honeydew from *Abies alba*, produced by bees towards the end of August, when no more flowers are available, from fir resin that had been already pre-digested by insects. Even in honeydew (in Italian “melata”), only five bee’s proteins out of the seven of all other types of honeys could be detected (unpublished experiments with Dr. M.M. Staver, University of Rijeka).

## 5. Analysis of Vegetable and Fruit Food Stuff

One of the first nourishment of plant origin we have studied is spinach, possibly under the influence of the Popeye saga. Fasoli *et al.* [40] mapped the cytoplasmic proteome of spinach leaves by capturing it at three pH values (4.0, 7.0 and 9.3) and eluting in 4% boiling SDS, 20 mM DTT. The number of gene products found exclusively thanks to the use of CPLLs and classified as low-abundance was particularly high, with 208 proteins *versus* 114 found in the initial extract with an overall gene product count of 322. Two main phenomena allowed detecting so many more proteins: the massive reduction of the concentration of RuBisCO and the enrichment of many rare proteins initially present in trace amounts made possible by the use of a relatively large initial volume of spinach extract. Among the enriched cellular components were ribosomal proteins, such as 30S, 40S, 50S and 60S. Other low-abundance proteins found in spinach leaves were those related to chloroplast category and those operating in the photosynthetic organelles, e.g., inorganic pyrophosphatase-1 and phosphoglycerate kinase. From the same category, but involved in the CO_2_ fixation and carbohydrate metabolism, were glutamate decarboxylase 2, glucose-1-phosphate adenylyltransferase, phosphoglucomutase, fructose bisphosphate aldolase 2, ribulose-phosphate 3-epimerase and sucrose-phosphate synthase.

The next proteome of importance in human nutrition we have analyzed is the one of olive pulp and seed (*Olea europaea* L.) due to the importance of this fruit in production of olive oil. Esteve *et al.* [41] have tackled this task via the CPLL approach. Thanks to its use, a quite large number of compounds has been indeed identified: 61 in the seed (*vs.* only four reported in current literature) and 231 in the pulp (*vs.* 56 described so far) [42]. In the seed, it highlights the presence of seed storage proteins, oleosins and histones (of which as many as 14 different unique species have been listed). In the pulp, the allergenic thaumatin-like protein (Ole e 13) was confirmed, among the other 231, as the most abundant protein in the olive fruit.

**Figure 2:**
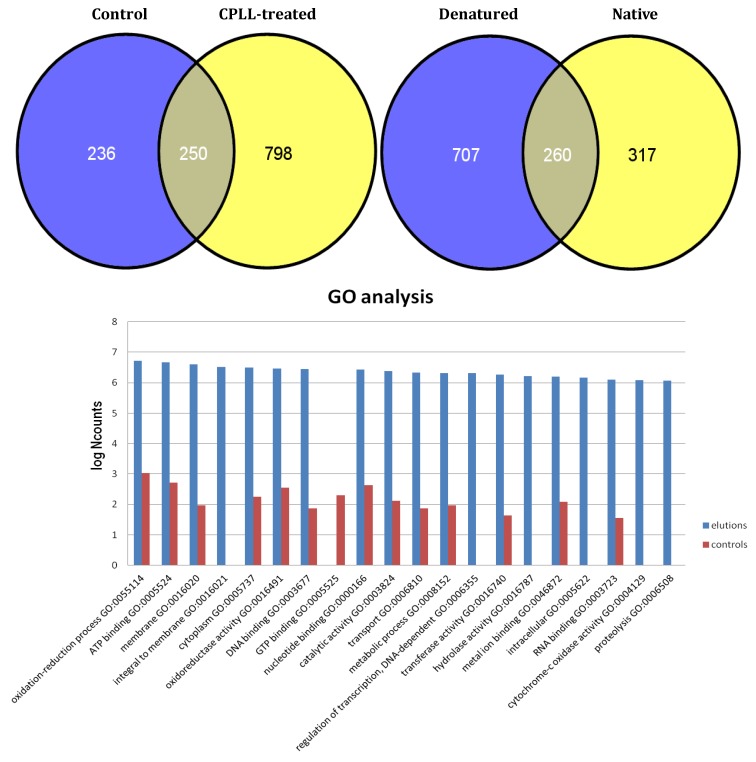
Upper panel: Venn diagrams showing the total identifications of avocado proteins in either the control or in the CPLL treated samples (left diagrams). The Venn diagrams to the right display the relative contributions to the total IDs of the denatured *vs.* the native sample extractions. Lower panel: GO analysis of the proteins detected in various metabolic processes in the control *vs.* CPLL-treated samples. In the latter case, five additional metabolic processes can be described, which are not visible in the control sample and most likely represent low- to very-low abundance proteins, whose visibility has been substantially enhanced via the CPLL technology (modified from Esteve *et al.* [43]).

Although the results on both systems were exciting, due to large increments of discovery as compared to published literature, yet we were annoyed by the fact that, in plant specimens, we did seem to be unable to break the barrier of 300 detected proteins, a much too low proteome harvest, considering that several thousand gene products should be present in these specimens too (although it should be noted that, up the these investigations, we only analyzed cytoplasmic soluble species, captured under native conditions). We thus turned to “recalcitrant” tissues, in search of ways and means to break these barriers. Two such tissues would certainly be the banana and avocado proteomes, since here tiny amounts of proteins (*ca.* 1%) would be dispersed into huge amounts of interfering plant matrices (polysaccharides and solid oils, respectively). These would be quite challenging projects and it was felt that one needed extra efforts to study these tissues and in general any plant proteome, efforts that have materialized in more than one extraction protocol, i.e., under native and denaturing conditions, in both cases followed by capture with CPLLs of the extracted proteins. The extraction under denaturing conditions was performed in 3% SDS, an anathema in CPLL treatments, since it would completely inhibit the capture. The SDS had to be removed either via the classical acetone/methanol precipitation, or by dilution from 3% to 0.1% in the presence of another compatible surfactant, like 1% CHAPS, conditions that would be compatible with the CPLL technology. The results in the case of avocado were outstanding: about 1300 unique gene products were detected, a four-fold increment as compared to the findings in olive pulp proteome. Figure 2 details the various discoveries obtained with the treatments: the unique species identified in the control were 236 *vs.* 796 in the global CPLL treatment, 250 being common in the two sets. The Venn diagram to the right shows how much more the denaturing conditions’ extraction contributed to the total discovery as compared to the native protocol: the unique catch has been more than double. The bar graph of GO analysis (lower panel in Figure 2) shows also some outstanding data: not only all the various Go categories are considerably more populated in the CPLL-captured samples, but also five new metabolic processes, not discernible in the control, could be described [43]. 

The next fruit investigated was the banana. *Musa* spp., comprising banana and plantain, is grown extensively in many developing countries and is considered to be one of the most important sources of energy in the diet of people living in tropical humid regions. Due to its antioxidant and cell antiproliferative activities, the consumption of banana has been associated with reduced risk of chronic diseases such as cardiovascular diseases and cancer [44]. Up to date, no in depth work has been focused on identifying the banana fruit proteome; since fresh banana pulp contains approximately 20% of carbohydrates and only 1% of proteins, this fruit has been traditionally considered as a difficult matrix for protein extraction, being a target in studies of optimization of protein extraction methodologies [45]. Fruits, as every biological source, contain highly-abundant proteins, which are often of limited interest for proteome analysis, whereas other proteins may be orders-of-magnitude less abundant, although still of high importance. Here too, by using CPLLs, advanced mass spectrometry techniques and *musa* mRNAs database in combination with Uniprot_viridiplantae database, we could identify, for the first time, 1131 proteins [46]. Among this large amount of species found, several already known allergens such as *musa a 1*, pectinesterase, superoxide dismutase and potentially new allergens have been detected. Additionally, several enzymes involved in degradation of starch granules and strictly correlated to ripening stage were identified. These results constitute the largest description so far of the banana proteome.

Spurred by these encouraging results, we decided to explore also the proteome of mango, a tropical fruit of very large consumption. In this case, we opted for analysis also of its peel (since also this part of the fruit is exploited for cooking) as well of the peptidome content (the latter as captured with a C_18_ resin). The aim of this study was not only to perform the deepest investigation so far of the mango proteome, but also to assess the potential presence of allergens and of peptides endowed with biological activities. The proteins of peel and pulp were captured under both native and denaturing extraction techniques and the harvest was outstanding. A total of 334 unique protein species have been identified in the peel *vs.* 2855 in the pulp, via capture with CPLLs at different pH values (2.2 and 7.2). In this very large pulp protein list, we could also identify some well-known allergens, in particular the non-specific lipid transfer protein, superoxide dismutase, germin-like protein and profilin [47]. 

## 6. Proteome Analysis of Non-Alcoholic Beverages

Here too, we will limit this survey to beverages that have been explored by the CPLL methodology, in comparison with untreated samples, as reported in the literature. We will only deal with proteomes, although in such beverages also a peptidome screening could be performed with CPLLs. The investigations reported below demonstrated the unique performance of CPLLs, such as: (1) the ability to handle large sample volumes (one liter and more) in an easy and user-friendly protocol; (2) sample enhancement factors up to four orders of magnitude; (3) extremely high detection sensitivities, reaching as little as 1 µg protein/L of sample, a sensitivity rarely obtained by present-day methodologies. Needless to say, these results were much superior than those obtained with conventional techniques, due to their inability to properly concentrate and “normalize” the relative concentration of the proteomes under analysis.

**Figure 3:**
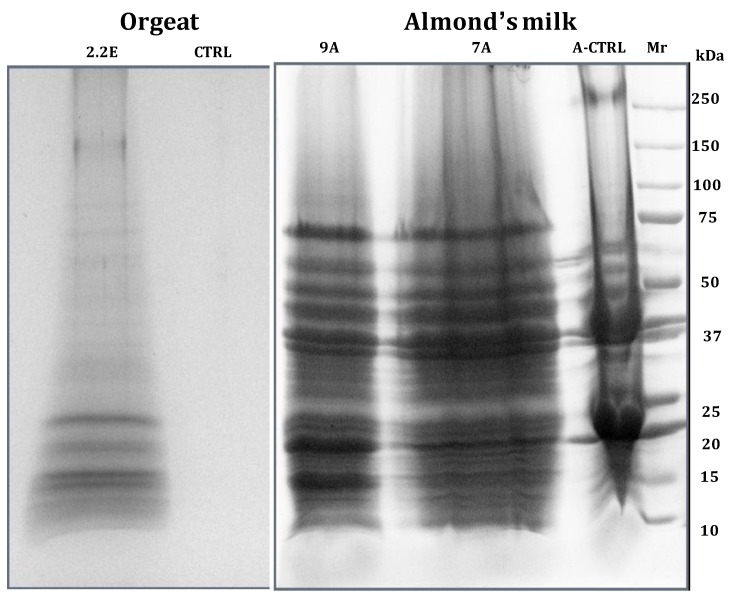
SDS-PAGE profiles of orgeat syrup (two tracks to the left) and of almond’s milk (right panel). Orgeat: CTRL = 100 μL of control orgeat solution before CPLL treatment. 2.2 E = 50 μL 2% SDS eluate of orgeat solution treated with a home-made-CPLL library at pH = 2.2. Almond’s milk: control (A-CTRL) and eluates from a 1:1 mixed library of PM and home-made-CPLLs at pH 7.2 (7A) and pH 9.3 (9A) values (modified from Fasoli *et al.* [48]).

There are at least two reasons for exploring the trace proteome of non-alcoholic beverages (whose proliferation in the shelves of supermarkets is appalling, with so many brands and fancy appellations as to stun the customers): (1) in order to certify the genuineness of such products and find out if they contain proteins of the vegetable extracts they have been prepared from; (2) in order to screen for the presence of any potential allergen in such beverages. Indeed, such analyses have been made on almond’s milk and orgeat syrup [48], coconut milk [49], as well as on a cola drink [50], ginger ale [51] and even white-wine vinegar [52]. In the case of almond’s milk, 137 unique protein species were identified, whereas in the case of orgeat syrup, a handful of proteins (just 13) were detected, belonging to a bitter almond extract. In both cases, the genuineness of such products was verified. On the contrary, cheap orgeat syrups produced by local supermarkets and sold as their own brands were found not to contain any residual proteins, suggesting that they were likely produced only with synthetic aromas and no natural plant extracts. Figure 3 displays the SDS-PAGE profiles of orgeat syrup (two tracks to the left) and of almond’s milk. In the case of orgeat, it can be appreciated that no single protein band is visible in the control, untreated sample; as for almond’s milk, whereas the control lane shows two major bands at 23 and 35 kDa, plus a few other zones, the two eluates after capture at pH 7.0 and 9.3 exhibit a very extensive band profile from 10 to 100 kDa. In the case of coconut milk, a grand total of 307 unique gene products could be listed, 200 discovered via CPLL capture, 137 detected in the control, untreated material and 30 species in common between the two sets of data. This is by far the most extensive mapping of this nutritious beverage, in which, up to the present, only a dozen proteins were known, those belonging to the high- to very-high abundance class.

An interesting detective story can here be told about the “invisible” proteome of a cola drink (nothing to do with Coca Cola, this one is an English beverage!) [50], stated to be produced with a cola nut extract. Indeed, a few proteins in the molecular mass (Mr) 15–20 kDa range could be identified by treating large beverage volumes (one liter) and performing the capture with CPLLs at very acidic pH values (pH 2.2) under conditions mimicking reverse-phase adsorption. It turned out that the proteins identified were barely three: one belonging indeed to the cola nut, the other two identified as agave species. Indeed, in this beverage label, it was stated that the cola drink had been generously sprinkled with 6% agave syrup, honor saved, all around. Conversely, things did not go so well (for the producer) in the case of a ginger beverage [51]. Although in traces, the presence of five grape proteins and one apple protein could be confirmed, but not even the faintest trace of any ginger root proteins. The first two findings are correct, as the producer stated that this beverage had been reinforced with 12% grape juice and 6% apple juice, but the absence of even traces of ginger proteins did not permit the classification of this beverage as a ginger extract on a proteomics scale. It was thus concluded that either the *Zingiber officinalis* was present in traces, or only its flavors had been added and not any root extract.

**Figure 4:**
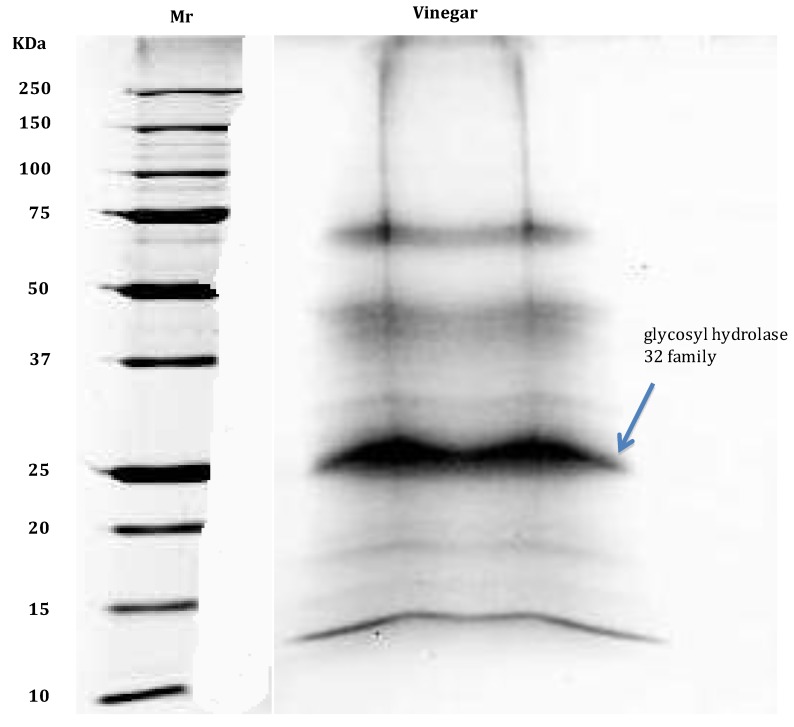
SDS-PAGE of white-vine vinegar. Mr: molecular mass ladder. Laemmli discontinuous buffer. Gel: 8%–18% polyacrylamide gradient. Run: three hours at 300 V. Sample load: 25 µL containing *ca.* 50 µg protein. Staining with micellar Coomassie Brilliant Blue. The major protein (the whole genome shotgun sequence of line PN40024, scaffold_22, a protein belonging to the glycosyl hydrolase 32 family) is indicated with an arrow (modified from Di Girolamo *et al.* [52]).

In another investigation on non-alcoholic beverages, the trace proteome of white-wine vinegar was identified again via CPLLs but under conditions mimicking reverse-phase capture, i.e., at pH 2.2 in presence of 0.1% trifluoroacetic acid. A total of 27 unique gene products could be tabulated, of which 10 specific of the database *Vitis vinifera*, 13 found in the general database Uniprot_viridiplantae and four in Swiss Prot_all entries. The most abundant species detected, on the basis of spectral counts, appears to be the whole genome shotgun sequence of line PN40024, scaffold_22 (a protein of the glycosyl hydrolase family, indicated by an arrow in Figure 4) [52]. The hypothesis set forward regarding these 27 surviving proteins to the strongly acidic, pH 2.2, environment of vinegar is that they could be the only ones resistant to denaturation, a phenomenon that typically occurs with proteins which have a tightly packed hydrophobic core and are thus insensitive to pH denaturation. The other interesting aspect of this set of 27 survivors is the fact that they seem to be resistant also to proteolytic attack, since their apparent Mr values (see Figure 4) are well distributed in the 70–120 kDa range. Conversely, when the ultra-trace proteome of a cola drink was explored, the minute traces of the only three surviving proteins were found solely in the leading/terminating boundary of the Laemmli discontinuous buffer in the SDS-PAGE gel, where typically only protein fragments are confined, suggesting that they were all degraded species.

## 7. Proteome Analysis of Alcoholic Beverages

Due to its extensive consumption worldwide, an investigation on wine proteome was surely due. To complicate the matters, though, modern wines might be quite different from those drunk by our ancestors. One of the main reasons is that the residual grape proteins, that survived the fermentation process, slowly aggregate leading to amorphous sediments or flocculates, causing turbidity. A haze or deposit in bottled wine indicates that the product is unstable, has a low commercial value and is therefore unacceptable for sale. For these reasons, it has become customary to remove the residual proteins remaining in the finished product, so as to prevent haze formation and sediment in the bottled wines available for sale. Among the fining agents, one of the most popular is casein derived from bovine milk, which, however, is a known allergen, whose presence should be declared on the label (something that no wine producer has ever done!). In fact, when an entire bottle of white wine (750 mL) was treated with CPLLs at pH 3.3, the results were quite exceptional, in that as little as 1 µg casein could be assessed as a residue after the treatment [53]. The major result of the above investigation was that it was proven that the CPLL technique had a sensitivity 200 times higher than the current ELISA test, a non-negligible accomplishment. In a competing study, Monaci *et al.* [54] stated “when fined wine samples were considered, the lowest added concentration for which the peptide marker could be detected was 50 μg/mL” (the peptide marker referring to casein digests, as identified by mass spectrometry), which seems to be a terribly low detection sensitivity, 50,000 times lower than that reported by Cereda *et al.* [53]. These authors, actually, continued in their search of traces of caseins in white wines and reported yet another method for tracing residual milk allergens, this time based on the use of a single-stage Orbitrap MS instrument [55]. Yet, the improvement in detectability was not spectacular, in their own words: “minimum detectable added caseinate concentrations were estimated between 39 and 51 µg/mL”. There was a further evolution on this topic. Palmisano *et al*. [56] published an extensive investigation on grape proteins present in white chardonnay. They adopted a multiplexed glycopeptide enrichment strategy in combination with tandem mass spectrometry in order to analyze the glycoproteome of this brand of white wine, thus identifying a total of 28 glycoproteins and 44 glycosylation sites. The identified glycoproteins were from grape and yeast origin. In particular, several glycoproteins derived from grape, like invertase and pathogenesis-related (PR) proteins, and from the yeast, were found after the vinification process. Bioinformatic analysis revealed sequence similarity between the identified grape glycoproteins and known plant allergens. These data, though, could be obtained only by analyzing a wine which had not been treated with any fining agent. Aware of that, D’Amato *et al.* [57] analyzed some bottles of Recioto wine (a dessert wine made from partly dehydrated grapes left for longer periods on the plants) and of Garganega wine, a white table wine produced from the same grapes picked at ripening, all of them not treated with any fining agent. A CPPL capture at four different pH values (pH 2.2, 3.8, 7.2 and 9.3) was performed. The combined data on the discoveries in the four CPLL eluates, as well as in the collected bottle sediment, allowed the identification of 106 unique gene products belonging to *Vitis vinifera*, as well as of an additional 11 proteins released by the *S. cerevisiae* used in the fermentation process. More recently, we have continued such analyses on champagne, produced in the Reims region: in aged champagne, 40 unique gene products could be detected for the first time [58]. 

The next logical step was to extend these investigations to red wines, here too either to detect traces of fining agents or the entire grape-proteomic asset in non-treated samples. In the case of red wines, one would expect to find ovalbumin or entire egg white, since these are the customary fining agents adopted. However, in an extensive investigation of Italian red wines, D’Amato *et al.* [59] found them to not contain traces of egg albumins but again of bovine caseins, especially α- and κ-caseins, with essentially no residual grape proteins, except for traces of thaumatin fragments. With another surprising finding: the wines analyzed (in North Italy, around the lake of Garda, 2009 harvest) contained (albeit in traces) abnormal amounts of proteins originating from fungal infection, such as from *Botryotinia fuckeliana*, *Sclerotinia sclerotiorum*, *Aspergillus aculeatus*, suggesting adverse meteorological conditions at harvest in that year. Traces of egg-white proteins in red wines were indeed detected Tolin *et al*. [60] but in wines that they had treated with different amounts of ovalbumin just to determine the detection limit of the MS analyses.

Following these extensive investigations of white and red wines, a study on the beer proteome was definitely due. This came from Fasoli *et al.* [61] who assessed this proteome again via CPLL capture at three different pH (4.0, 7.0 and 9.3) values. Via mass spectrometry analysis of the recovered fractions, after elution of the captured populations in 4% boiling SDS, they could categorize such species in 20 different barley protein families and two maize proteins, the only ones that had survived the brewing process (the most abundant ones being *Z*-serpins and lipid transfer proteins). In addition to those, they could identify no less than 40 unique gene products from *Saccharomyces cerevisiae*, as routinely used in the malting process. These latter species must represent trace components, as in previous proteome investigations barely two such yeast proteins could be detected.

The natural extension of these investigations on the proteome of alcoholic beverages was to explore the trace proteome of aperitifs, i.e., alcoholic beverages half a way in between wines (with a 11%–13% alcohol content) and hard liquors (e.g., cognac, whiskey, grappa and the like, typically with more than 40% alcohol). Aperitifs generally have an alcohol content around 20%–22% (although their alcohol content can be lower, down to *ca.* 15%) and are in general made with herbal infusions. Their widespread popularity stemmed from their large consumption in pubs and social gatherings and from the medical observation that they would favor food intake and stimulate digestion (these bitter liquors were classified as cholagogues, i.e., medicinal agents promoting the discharge of bile from the system). Since in general all producers declare that their aperitifs are made with herbal infusions, it was of interest to investigate their trace proteome, in order to verify the genuineness of such products. With this in mind, Fasoli *et al.* [62] decided to analyze a popular aperitif in North Italy, Amaro Braulio, in search of residual proteins, if any. This aperitif is made with an infusion of 13 mountain herbs and berries, among which four are officially indicated in the label: *Achillea moschata*, juniper (*Juniperus communis* subsp. *alpina*) berries, absinthe (*Artemisia absinthium*) and gentian (*Gentiana alpina*) roots. Via capture with solid-phase combinatorial peptide ligand library at pH 7.0 and 2.2 these authors were able to identify 72 unique gene products, among which the PR5 (parasite resistance) allergen *Jun r 3.2*, a 25 kDa species from *Juniperus rigida*. Due to the paucity of data on these alpine herbs, it was difficult to attribute these proteins to the specific plant extracts presumably present in this beverage; however, most of the species identified indeed belong to alpine herbs and plants, living in a habitat between 1000 and 2000 m of elevation. Most of them are enzymes, spanning a Mr range from 10 to 65 kDa. Thus, the genuineness of this product was amply proven. Things did not go so well, though, with another popular aperitif sold the world over, Cynar, stated to be made with an infusion of artichoke leaves reinforced too with an extract of 13 secret herbs. To err on the safe side, Saez *et al.* [63] adopted a three-pronged attack to tackle the issue. First, different extraction techniques were used for the characterization of the artichoke’s proteome, secondly a home-made infusion was analyzed and finally the proteome of the commercial drink was checked. The artichoke proteome was evaluated via prior capture with CPLLs at four different pH (2.2, 4.0, 7.2 and 9.3) values. Via mass spectrometry analysis of the recovered fractions, after elution of the captured populations in 4% boiling SDS, these authors could identify a total of 876 unique gene products in the artichoke extracts, 18 in the home-made infusion and no proteins at all in the Italian Cynar liqueur, casting severe doubts on the procedure stated by the manufacturer (that should be made by an infusion of artichoke leaves plus 13 different herbs). It would thus appear that CPLLs could be a formidable tool for investigating the genuineness and natural origin of all commercial drinks in order to protect consumers from adulterated products.

As we are Italians, we could not possibly terminate this review without investigating the proteome of another very popular digestive, namely Limoncello, stated to be an infusion of the outer lemon peel, the flavedo. We took this occasion to investigate too the proteomes of lemon peels and pulp, of a home-made alcoholic infusion and finally, to close the circle, of commercial liqueurs. The aim of this study was not only to perform the deepest investigation so far of the lemon peel proteome but also to assess the genuineness of the commercial liqueur via a three-pronged attack. First, different extraction techniques have been used for the characterization of the peel (and additionally of the pulp) proteome, secondly a home-made infusion was analyzed and finally the proteome of the commercial drink was checked. The peel (the flavedo, not the underlying layer called albedo) proteome was evaluated via prior capture with CPLLs at different pH values (2.2 and 7.2). Via mass spectrometry analysis of the recovered fractions, after elution of the captured populations in 4% boiling SDS, we could identify a total of 1011 unique gene products in the peel extracts and 674 in the pulp, 264 proteins in the home-made infusion and just eight proteins (and protein fragments), together with 12 peptides, in one Italian Limoncello produced in the Sorrento Region, thus proving the genuineness of this product [64]. On the contrary, cheaper Limoncellos were devoid of any protein/peptide, casting doubts on their production from vegetable extracts. Thus, it would appear that CPLLs could prove a powerful technique for investigating the genuineness and natural origin of commercial drinks in order to protect consumers from adulterated products.

## 8. Conclusions: Mammalian *versus* Plant Proteomics

**Figure 5:**
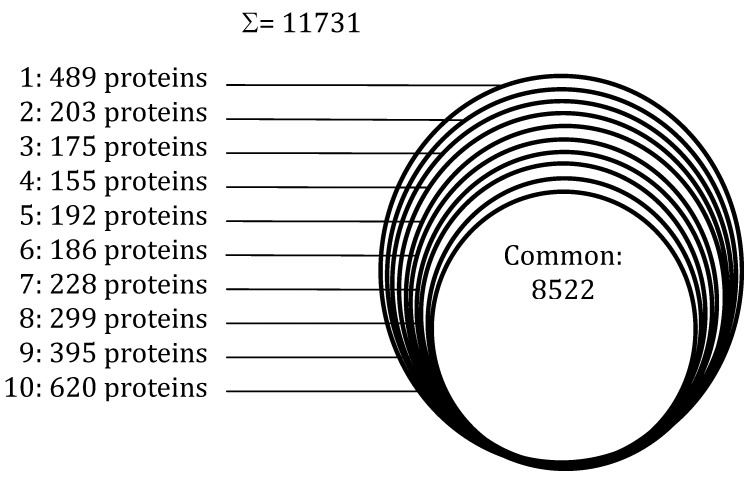
Venn diagrams of the proteins identified in 10 different human cell lines. Each cell exhibited an average of 10,361 species, of which 8522 proteins represented a common set and the remaining a set specific for each cell line, as indicated (modified from Geiger *et al.* [65]).

When surveying the deeds of scientists working with mammalian proteomics, one can find that today they can explore to an incredible extent the proteome of any living cell line, as shown in Figure 5. When analyzing 11 human cell lines, Geiger *et al.* [65] could identify a total of 11,731 proteins and on average 10,361 ± 120 proteins in each cell line, an outstanding catch, indeed. Interestingly, a very large number of them represent a common set shared by all cell lines, amounting to 8522 unique species. Each individual cell then displays from 200 to 500 proteins specific of each line. The latter probably represent proteins that characterize each individual line and ensure its specific biological activity. Likely, they could also be low-abundance species. How can there be such a discrepancy with plant proteomics, when in this last domain we are lucky if we can find a few hundred species in a single run? It should be noted that, in a way, these mammalian cell lines grown in *in vitro* cultures are rather “easy” samples, in that they are not embedded in, e.g., fibrous tissues, muscles and other body compartments that would represent a complex matrix from which such cells would have to be extracted. On the contrary, in plant proteomics, most of the time the proteins to be identified are dispersed into a very complex matrix and often are present in low amounts as compared to the plant biopolymers (e.g., polysaccharides, polyphenols) and metabolites constituting the specimen mass. This is the reason why, most of the time, it has been difficult to detect more than a few hundred species in any plant tissue. Thus, the CPLL methodology here described, allowing access to an order of magnitude more so (up to 3000 unique gene products) represents an important advance in the field. Clearly, however, more efforts should be dedicated to improving extraction technologies so as to match what today is obtained in mammalian proteomics.
